# Large-scale pharmacological profiling of 3D tumor models of cancer cells

**DOI:** 10.1038/cddis.2016.360

**Published:** 2016-12-01

**Authors:** Lesley A Mathews Griner, Xiaohu Zhang, Rajarshi Guha, Crystal McKnight, Ian S Goldlust, Madhu Lal-Nag, Kelli Wilson, Sam Michael, Steve Titus, Paul Shinn, Craig J Thomas, Marc Ferrer

**Affiliations:** 1 National Center for Advancing Translational Sciences, Division of Pre-Clinical Innovation, National Institutes of Health, Bethesda, MD, USA

## Abstract

The discovery of chemotherapeutic agents for the treatment of cancer commonly uses cell proliferation assays in which cells grow as two-dimensional (2D) monolayers. Compounds identified using 2D monolayer assays often fail to advance during clinical development, most likely because these assays do not reproduce the cellular complexity of tumors and their microenvironment *in vivo*. The use of three-dimensional (3D) cellular systems have been explored as enabling more predictive *in vitro* tumor models for drug discovery. To date, small-scale screens have demonstrated that pharmacological responses tend to differ between 2D and 3D cancer cell growth models. However, the limited scope of screens using 3D models has not provided a clear delineation of the cellular pathways and processes that differentially regulate cell survival and death in the different *in vitro* tumor models. Here we sought to further understand the differences in pharmacological responses between cancer tumor cells grown in different conditions by profiling a large collection of 1912 chemotherapeutic agents. We compared pharmacological responses obtained from cells cultured in traditional 2D monolayer conditions with those responses obtained from cells forming spheres *versus* cells already in 3D spheres. The target annotation of the compound library screened enabled the identification of those key cellular pathways and processes that when modulated by drugs induced cell death in all growth conditions or selectively in the different cell growth models. In addition, we also show that many of the compounds targeting these key cellular functions can be combined to produce synergistic cytotoxic effects, which in many cases differ in the magnitude of their synergism depending on the cellular model and cell type. The results from this work provide a high-throughput screening framework to profile the responses of drugs both as single agents and in pairwise combinations in 3D sphere models of cancer cells.

Many new cancer drug candidates are being identified using cancer cell lines in conjunction with cell proliferation assays where cells are cultured as a two-dimensional (2D) monolayer of cells on plastic surfaces. Although technically very amenable to screening large collections of compounds, cells grown under these conditions do not render the same cell–cell interactions and thus are not subject to the same microenvironment as cancer cells in a tumor *in vivo*. As a consequence, cells are likely to be metabolically and genetically different, and therefore respond differently to pharmacological agents. For many years now spheroid models have been developed in an attempt to mimic the architecture, cellular contacts, cellular heterogeneity, metabolic, genetic and differentiation state of cells in tumors and the subsequent effects of the tumor microevironment.^1, 2^ These three-dimensional (3D) models of tumors range in complexity from layered cellular systems, to single-cell type spheres of different sizes, to complex multi-cell type spheres.^3, 4, 5, 6, 7^ 3D cultures are also being explored as models of tumor cell sub-populations called cancer stem cells (CSCs) or tumor-initiating cells (TICs).^8^ CSCs cells are thought to drive metastasis and tumor formation, and to be resistant to current chemotherapy and radiation therapy treatments leading to cancer recurrence.^9, 10, 11, 12^ The discovery of chemotherapeutics that potently induce CSCs cell death is therefore of high interest to develop more efficacious anticancer therapies that address recurrence and metastasis.

The different 3D tumor models available have been challenging to use for large-scale drug screening because of the difficulty in generating consistent and reproducible results in microtiter plates used for high-throughput screening (HTS). We have been developing methodologies to produce 3D sphere cultures in a 1536-well format to enable the HTS of large collections of small molecules. In this regard, we have previously reported a limited 1536-well cell proliferation screen in culture conditions where cells were in the process of forming spheres,^13^ previously shown to be enriched for cells with high expression of stem cell markers.^14, 15^ Here, we expand that work by using a newly developed method to produce and screen 3D spheres in a 1536-well microplate format. We used the *KRAS* mutant pancreatic cancer cell line PANC1 and the kidney cancer line SN12C, both of which have been shown to develop CSC-enriched 3D spheres.^13, 14, 15, 16, 17, 18^ Both pancreatic and kidney cancers are aggressive, develop metastatic tumors and have characteristic markers of CSCs with very few treatment options. Using these newly developed HTS amenable assays, we screened an oncology-focused, mechanistically annotated library of 1912 chemotherapeutic agents^19, 20, 21^ to find new drugs and/or drug combinations that cause death of these cells in 3D spheres or cells forming spheres. This library embraced mechanistic redundancy for the mechanism of action of the compounds, thus enabling the analysis of the results for target and pathway enrichment.

## Results

### Development of a 1536-well microplate 3D spheroid cell proliferation assay

**Figure 1:**
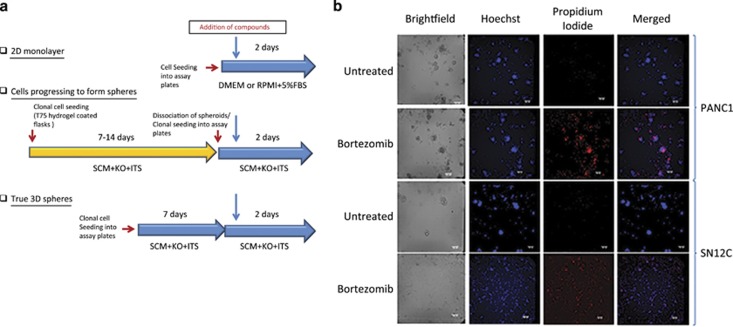
PANC1 and SN12C cell cultures in 2D monolayer, forming spheres and 3D spheres. (**a**) Adherent PANC1 cells were seeded in DMEM containing 10% FBS and SN12C in RPMI with 10% FBS into solid bottom white tissue culture treated 1536-well plates. Spheroid derived cells were initially cultured for 7-14 days in ULA flasks in SCM and then dissociated with Trypsin and plated into the same 1536-well assay plates as above in SCM. 3D spheres were formed by growing either PANC1 or SN12C in SCM for 7 days in the 1536-well tissue culture plates using an adhesive top seal to prevent evaporation while the spheres were forming. All three modes of cell growth were treated with the compounds for 48 h. Cell viability was quantitated using the CellTiterGlo^TM^ reagent. (**b**) 3D spheres of PANC1 and SN12C cells were formed in wells of a 1536-well microtiter plate seeding and incubating cells for 7 days in SCM. The morphology of the spheres was determined using brightfield and fluorescence imaging. In all, 4x images were captured of a whole well using an Incell2000 High Content Reader (GE Healthcare, Marlborough, MA, USA). Hoescht dye was used to stain all cells and PI was used to stain for dead cells

3D spheres were formed from the PANC1 and SN12C cell lines in each of the wells of a 1536-well microtiter plate when grown in a defined growth media called stem cell media (SCM; Figure 1a). After 7 days, we observed the formation of spheres of up to ~100 *μ*m in diameter for both PANC1 and SN12C cells (Figure 1b). On average, each well contained ~20–30 spheres of 50–100 *μ*m in diameter (data not shown). After treatment with a toxic proteasome inhibitor bortezomib, all cells were stained with Hoechst and dead cells with propidium iodide (PI). Our data demonstrated that these spheres were not large enough to have a hypoxic core of dead cells, like seen in other spheroids grown under 384-well conditions. The overall plate statistics for the cell viability assay (as measured with the CellTiterGlo reagent) for 3D spheres grown in 1536-well plates demonstrated that the assay was robust for use in HTS with a coefficient of variation of 7% and Z'-factor of 0.7^22^ (Supplementary Figure 1).

### Dose-response quantitative screening of the mechanism interrogation PlatE (MIPE) oncology collection

For the dose-response screening, the compounds were tested at 11 doses, starting at 46 *μ*M, and diluted three-fold to generate dose responses. Data were analyzed using the curve response class (CRC) algorithm developed at NCATS for qHTS data.^23, 24^ This analysis produces several curve responses parameters, including a CRC score for the overall quality of the dose response (overall measure of potency and efficacy), an IC_50_ (potency), as well as a % activity at the maximum concentration tested (MAXR). The results from the CRC analysis for each compound in each cell line and under each cell culture mode screened are found in an excel file in the Supplementary Material section.

**Figure 2:**
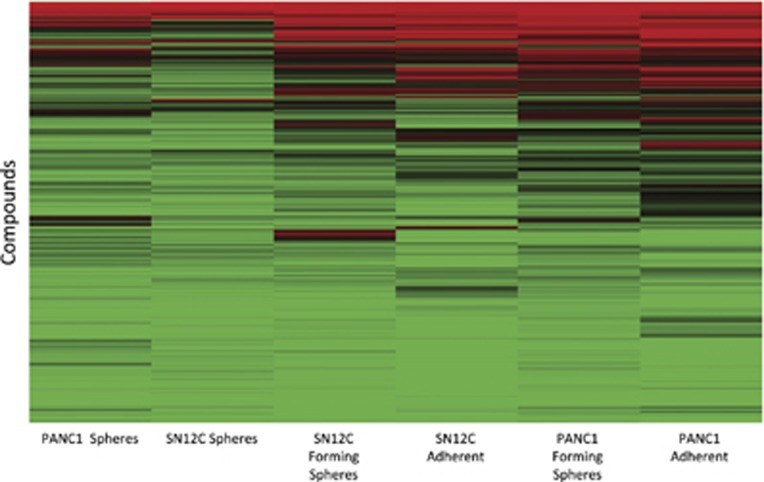
Pharmacological responses of the MIPE collection in PANC1 and SN12C in 2D monolayer, 3D spheres and cells forming spheres. Heat map of hierarchal clustering of % activity for each compound at the maximum concentration tested (46 *μ*M). The clustering displays both cancer types in all three growth modes, differentiated cells in 2D monolayer, cells forming spheres, and 3D spheres. Gradient of colors from red (0% viability), black (50% viability) and green (100% viability). Clustering was done using the Hierarchical clustering function in TIBCO Spotfire

Hierarchical clustering using MAXR (Figure 2) shows that the pharmacological responses of the compounds from the MIPE collection in cells growing in 2D monolayer and forming spheres clustered by cancer cell type, while the responses for 3D spheres cluster together for both cell lines. The heat map also shows that compounds that were more efficacious at reducing % viability in 2D monolayers had less activity in cells forming spheres, and had the least activity in 3D spheres.

### Identification of pan-active compounds

**Figure 3:**
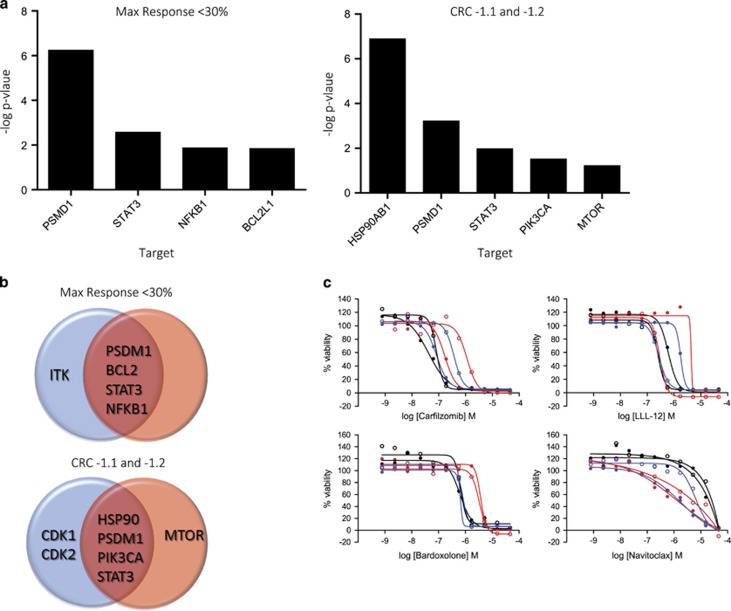
Selection of pan-active compounds. (**a**) Target enrichment plot for pan-active hits selected by MAXR <30% for both cell lines and all assay modes (left panel) and for pan-active hits selected by CRC −1.1 or −1.2 for both cell lines and all assay modes (right panel). -Log *P*-values were calculated as described in materials and methods based on the total number of compounds targeting a gene or mechanism. A –log *P*-value >1 was used as a cut-off to consider a target or processed being overrepresented. (**b**) Venn diagrams of the target classes enriched in the pan-actives compounds by cell line (red circle for PANC1 and blue circle for SN12C) using MAXR (top panel) and CRCs −1.1 and −1.2 (bottom panel). (**c**) Dose responses of selected representative compounds for the four most enriched target classes by the max response method: (•) PANC1 differentiated 2D monolayers, (•) PANC1 3D spheres, (•) PANC1 forming spheres, (○) SN12C differentiated 2D monolayers, (○) SN12C 3D spheres and (○) SN12C forming spheres

From the perspective of discovering new treatments against pancreatic and kidney cancer, we were interested in finding compounds with a strong cytotoxic effect in both cell types and in all cell culture modes. As the screen was implemented in a dose-response mode, results were analyzed using MAXR (measure of the efficacy of the compound) and the CRC scores, which is a measure of both compound efficacy and potency.^13^ A stringent cut-off of ≤30% viability was chosen to select active compounds by MAXR in all cells and assay conditions tested. Sixty-one compounds were identified as pan-active, and a target enrichment analysis showed a statistical (*P*-value <0.05) enrichment of pathways related to inhibition of BCL2, the proteasome, NF*κ*B and STAT3 (Figure 3a, left plot). These same targets were also enriched when each cell line was analyzed separately, as shown in the Venn diagrams in Figure 3b (top panel) and Supplementary Figure 2. Examples of dose responses from the primary quantitative HTS (qHTS) representing compounds from each one of these target classes are shown in Figure 3c.

A drawback of the traditional hit selection method based on a single % viability parameter to measure compound activity from HTS is that it selects compounds based solely on efficacy, and does not efficiently discern differential activity of the compounds based on potency. As our data generated dose responses in the primary HTS, we were able to assign a CRC to each compound.^25^ The CRC is a parameter that includes potency, efficacy and reliability of the compound effect. For the purposes of this study, we only considered active compounds those with high-quality dose responses (CRC −1.1 and −1.2). This includes potent compounds with full dose responses, but that may vary in the degree of efficacy at the maximum doses tested (−1.1 having full efficacy and −1.2 having partial efficacy). There are much fewer compounds with CRC −1.1 and −1.2 in the 3D sphere assay, regardless of cell line, than for the respective 2D adherent monolayer populations of the two cells lines (Supplementary Table 1). Target enrichment analysis of the 41 pan-active compounds with CRCs of −1.1 and −1.2 shows that the statistically significant overrepresented targets were HSP90, the proteasome, STAT3, mTOR and PI3K (Figure 3a, right plot). When the data were analyzed by cell line, pathways involving HSP90, the proteasome, STAT3 and PI3K were again identified as overrepresented (*P*-value <0.05) for both lines, whereas CDK1 and CDK2 were enriched targets selectively for PANC1, and mTOR inhibitors for SNC12C. Overlaps between cell lines in the different cell growth conditions are visualized in Venn diagrams (Figure 3b, bottom panel) and in Supplementary Figure 2.

### Compounds with differential activity between 2D monolayer and 3D spheroid cultures

**Figure 4:**
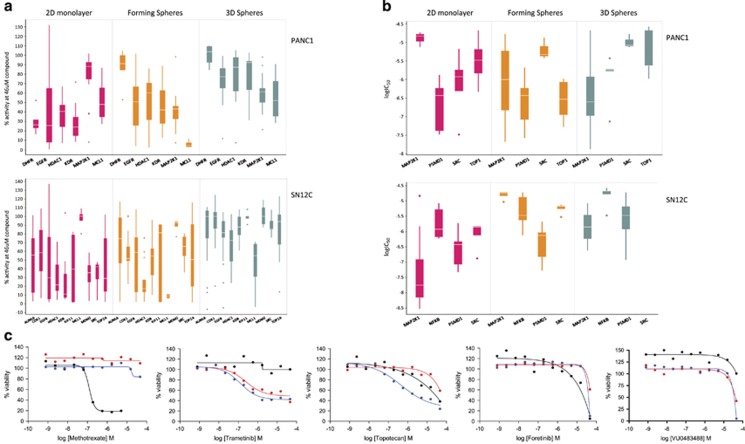
Selection of differentially active compounds. (**a**) Differentially active compounds from 2D monolayer growth mode by MAXR. Median MAXR was calculated for compounds in each target class and a *P*-value was computed for the differences in median MAXR between target classes in each mode growth. Box plots showing the distribution of MAXR for compounds in each target class differentially active (difference in median activity >40%, *P*-value<0.01) by maximum responses in the different assays modes for PANC1 cells. Top panel is for PANC1 cells and bottom panel for SN12C cells. (**b**) Differentially active compounds from differentiated 2D monolayer growth mode by potency. Compounds with CRC −1.1, −1.2, v2.1 and −2.2 were selected and a median log IC_50_ calculated for compounds in each target class and a *P*-value was computed for the differences in median AC50 values between target classes in each mode growth. Box plots showing the distribution of log IC_50_ values for compounds in each target class differentially active by fold change in median AC_50_ >10-fold and *P*-value<0.05 in the different assays modes for PANC1 cells (top panel) and SN12C cells (bottom panel). (**c**) Dose responses of selected representative compounds for the four most enriched target classes with differential activity between growth modes by the max response method (Methrotraxate for PANC1, Trametinib for PANC1, and VU0483488 for SN12C) and by differences in logIC_50_ (Topotecan for PANC1 and Foretinib for SN12C). (•)2D monolayers, (•) 3D Spheres, (•) cells forming spheres

Our analysis included mining for compounds which demonstrated differential activity under the different assay modes and cell lines. Compounds were selected based on differences in the median MAXR and logIC_50_ (restricted to those compounds producing complete dose responses as determined by CRCs −1.1, −1.2, −2.1 and −2.2) of all the compounds within a target class, and then we determined the statistical significance of the difference between these responses in the two cell lines and under the three growth modes. For this analysis, we chose to use a difference in the median of the MAXR of >40% and a *P*-value <0.01 to prioritize those target classes with larger and most robust differential activity by maximum response in Figure 4a (efficacy); and in Figure 4b, a difference of 10-fold in log IC_50_ with a *P*-value <0.05 for differences in potency. By using these criteria, for PANC1 cells, there were four target classes that were significantly more efficacious in 2D monolayer cultures than 3D spheres (DHFR, KDR, EGFR and HDAC1); and although they did not meet the criteria of >40% difference in MAXR, TUBB, AURKA and FAS showed very statistically significant (*P*-value<0.002) differences in MAXR. No target classes were significantly more efficacious in 3D spheres than 2D monolayers by differences in MAXR. In the top panel of Figure 4a, the box plots of the four targets with the largest differential activity between PANC1 cells grown in 2D monolayers *versus* those grown as 3D spheres are shown. Similarly, for SN12C cells, there were eight target classes that were significantly more efficacious in 2D monolayer cultures than 3D spheres (KDR, TOP2A, KIF11, EGFR, HDAC1, AURKA, SRC and CDK1). In addition, although they did not meet the criteria for difference by MAXR, TUBB, MET and PI3KCA and TOP1 were statistically significantly (*P*-value <0.002) more efficacious for 2D monolayers than for 3D spheres using MAXR (data not shown). MCL1 was the only target class that was statistically significantly (*P*-value <0.05) more efficacious (>40% MAXR) in 3D spheres than in 2D monolayers (Figure 4a, bottom panel). When looking at differential effects by potency, only one target class, MAP2K1, was significantly more potent for PANC1 3D spheres than 2D monolayers (Figure 4b, top panel), and inhibitors of NFκB were significantly more potent for SN12C 2D monolayers than 3D spheres (Figure 4b, bottom panel). Figure 4c shows dose responses for selected compounds with differential activity between 2D monolayers and 3D spheres.

### Compounds with differential activity between cells growing into 2D monolayer and cultures forming spheres

Using a similar analysis method as described in the previous section, one target class, DHFR, was significantly more efficacious for 2D monolayer cultures by difference in MAXR than for cultures forming spheres in the PANC1 cell line; and MCL1 and MAP2K1 were significantly more efficacious in cultures forming spheres than 2D monolayers for SN12C cells. In Figure 4a, the top panel shows box plots of selected targets with largest differential activity between PANC1 cells grown in 2D *versus* cultures of cells forming spheres. For SN12C cells, one target, MDM2, was significantly more efficacious in 2D monolayer cultures than cultures forming spheres. For PANC1, two targets, MAP2K1 and TOP1, were more potent in cultures forming spheres than 2D monolayers (Figure 4b, top panel); and MAP2K1 and SRC inhibitors were more potent for SN12C in 2D monolayers than cultures forming spheres (Figure 4b, bottom panel). Figure 4c shows dose responses for selected compounds with differential activity between 2D monolayers and cells forming spheres.

### Identification of compound combinations with enhanced cytotoxic effects in 3D spheroid cultures

**Figure 5:**
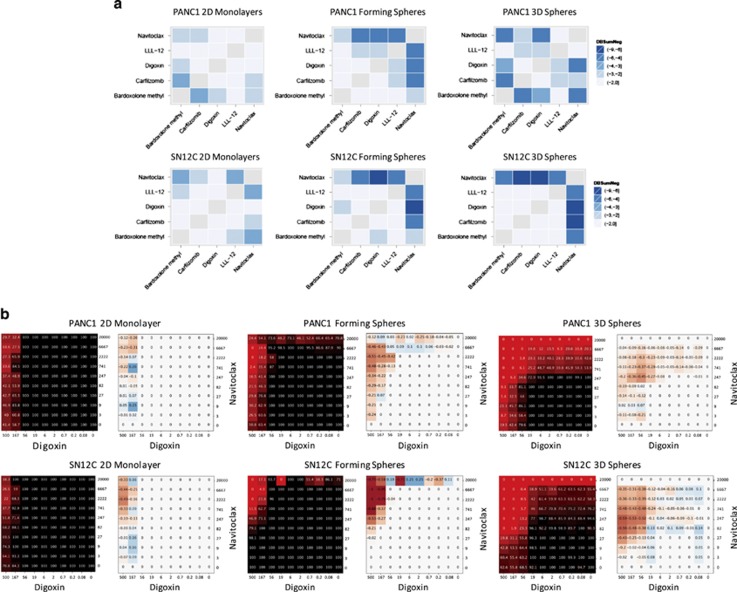
Enhanced inhibition of cell proliferation by combinations of selected compounds. (**a**) Heat map plots of the sum of negative delta bliss values for each combination dose-response matrix of the selected compounds for PANC1 (top panel) and SN12C (bottom panel) cells in each growth conditions. Darker blue values indicate higher synergy by sum negative delta bliss score. (**b**) Heat map plots of % activity and delta bliss scores for combination dose-response matrices of Navitoclax and Digoxin in PANC1 cells (top panels) and SN12C cells (bottom panels) grown in 2D monolayers, 3D spheres and cells forming spheres. For % activity plots, black refers to 100% viability and red to 0% viability. For delta bliss plots, higher negative values (in red) reflect higher synergy, whereas positive values (blue) refer to antagonism. Compound concentrations on the axis are expressed in nM

Representative compounds from the target classes that were found to be enriched as pan-active cytotoxic drugs were tested in pairwise combinations in the different growth modes using the combination screening platform previously described.^19, 20, 21^ The compounds tested included Carfilzomib (a proteasome inhibitor), Bardoxolone methyl (a KEAP inhibitor that inhibits the NFkB pathway), Navitoclax (a BCL2 inhibitor) and LLL-12 (a STAT3 inhibitor) (see Figure 3c for single-agent dose responses in all cell assay modes). In addition, one of the most potent and pan-active hits from the screens was Digoxin, an approved drug of the cardiac glycoside (CG) class (Supplementary Figure 3), and was also included in the combination screening. The pairwise matrix combination screen for these five compounds for both cell lines, in all growth modes, was done in replicates. Table 1 summarizes the results as the average of the sum of the negative delta bliss values for each pairwise compound combination. The same values are displayed as correlation plots in Figure 5a to illustrate the similarities and differences in synergism for the combinations of these five compounds for each cell line and culture mode tested. An arbitrary cut-off of <−3 sum of negative delta bliss values was chosen to select those combinations that were more synergistic. Interestingly, our data show that in general, there were more synergistic combinations in 3D spheres compared with cells forming spheres or the 2D cultures. Combinations of Navitoclax appear to drive synergy in 3D spheres and cells forming spheres, for both PANC1 and SN12C cells. Five combinations were very synergistic for PANC1 3D spheres, including Navitoclax and Bardoxolone methyl (average DBSumNeg −5.5); Navitoclax and Digoxin (average DBSumNeg −4.7); Carfilzomib and Bardoxolone methyl (average DBSumNeg −4.6); Digoxin and Bardoxolone methyl (average DBSumNeg −3.9); and LLL-12 and Digoxin (average DBSumNeg −3.0). Similarly, for SN12C 3D spheres, the four strongest synergies included Navitoclax and Digoxin (average DBSumNeg −8.7); Navitoclax and Carfilzomib (average DBSumNeg −6.3); Navitoclax and LLL-12 (average DBSumNeg −6.0); and Navitoclax and Bardoxolone (average DBSumNeg −5.5). Once again, Navitoclax appears to be driving synergies in cultures of cells forming spheres for both PANC1 and SN12C, and three combinations were highly synergistic in PANC1 cells growing to spheres, including Navitoclax and LLL-12 (average DBSumNeg −5.1); Navitoclax and Digoxin (DBSumNeg −4.3); and Navitoclax and Carfilzomib (DBSumNeg −4.1). For SN12C, the same three combinations were also highly syngeristic, Navitoclax and Digoxin (average DBSumNeg −7.6); Navitoclax and Carfilzomib (avergae DBSumNeg −5.0); and Navitoclax and LLL-12 (average DBSumNeg −4.7). Synergies are not as strong in 2D monolayer cultures as for 3D spheres and cells forming spheres. For PANC1, Carfilzomib and Bardoxolone (average DBSumNeg −3.7) is the only combination with synergy above the cut-off, and for SN12C, three combinations, Navitoclax and Bardoxolone (average DBSumNeg −3.5), Navitoclax and LLL-12 (average DBSumNeg −3.3), and LLL-2 and Bardoxolone (average DBSumNeg −3.0), again highlighting BCL2, NFkB and STAT3 pathways as being critical for SN12C cell survival. Figure 5b shows the heat maps of % activity and delta bliss score for the combination of Digoxin and Navitoclax, which was strongly synergistic in 3D sphere cultures on both PANC1 and SN12C cells.

**Figure 6:**
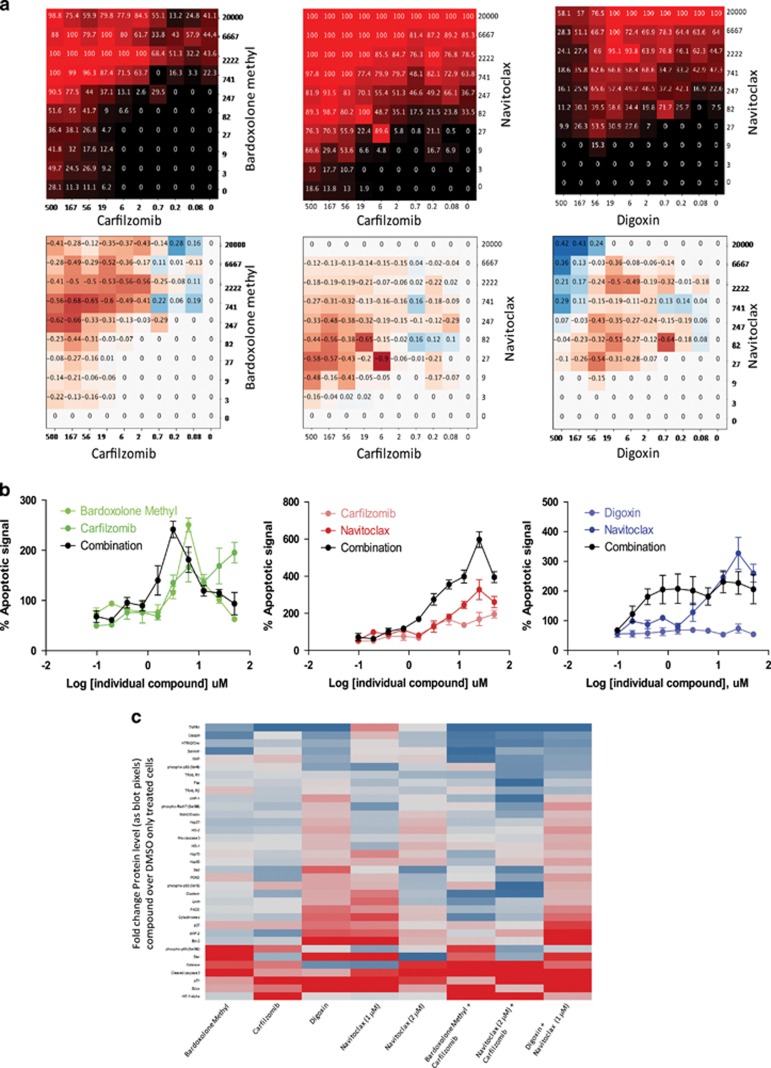
Enhanced induction of apoptosis by combinations of selected compounds. (**a**) Heat map plots of % activity and delta bliss scores for combination dose-response matrices of the three most synergistic compound combinations in the CaspaseGlo apoptosis cell death assay in PANC1 3D sphere cell culture assay. Top panels correspond to the % activity plots; black refers to 100% viability and red to 0% viability. Bottom panels are the delta bliss plots; higher negative values (in red) reflect higher synergy, whereas positive values (blue) refer to antagonism. Compound concentrations on the axis are expressed in nM. (**b**) Dose-response plots of % apoptosis measured with CaspaseGlo reagent after 20h treatment of individual and combinations on PANC1 3D spheres generated in a well of a 384-well ULA round bottom plate. (**c**) Heat map of the ratio of pixel intensity from blot dots on the apoptosis protein arrays (*y* axis) after after 20h treatment of individual and combinations, relative to treatment with DMSO, on PANC1 3D spheres generated in a T75 flask. Red indicates upregulation and blue indicates downregulation

We further explored whether three selected combinations with strong synergisitic effects on cell proliferation in 3D spheres (Carfilzomib and Bardoxolone, Navitoclax and Carfilzomib, and Navitoclax and Digoxin) induced apoptotic cell death in PANC1 3D spheres. We first tested the compounds in a dose-response pairwise combination matrix in 1536-well format, similar to the protocol described above for the cell proliferation assay. Second, we tested whether there was an enhanced induction of apoptosis in fixed ratio dose-response combinations in 3D spheres generated in 384-well plates, which are larger in diameter (approximately 400 *μ*m in diameter) than those generated in 1536-well plates (50–100 *μ*m) (see Supplementary Figure 4). Finally, we investigated the mechanism of apoptosis induction by measuring the regulation of apoptosis related proteins using antibody arrays from 3D spheres generated in flasks. These spheres are approximately 200 *μ*m in diameter (see Supplementary Figure 4). We found that the combinations Carfilzomib and Bardoxolone (average DBSumNeg −16.1), Navitoclax and Carfilzomib (average DBSumNeg −12.7), and Navitoclax and Digoxin (average DBSumNeg −9.0) were also strongly synergistic in 1536-well 3D sphere dose-response matrix apoptosis assay (Figure 6a). The apoptosis data measured in 3D spheres generated in a 384-well microplate showed induction of apoptosis by Navitoclax, Carfilzomib and Bardoxolone methyl, but not Digoxin, as early as 16 h (Figure 6b and supplementary Figure 5). Figure 6b also shows that a large enhanced induction of apoptosis is observed for the three combinations at 20h treatment, compared with 16 and 24 h (see supplementary Figure 5) for concentrations ranging from 1 to 4 *μ*M. The results of the apoptosis protein array are shown in Figure 6c (also in Supplementary Figure S6 and Supplementary Table S2) as a heat map of fold change in apoptosis protein levels for individual drug and drug combination treatments compared with DMSO treatment. Treatments were on the PANC1 3D spheres, after 20- h incubation with individual compounds and the corresponding compound combinations. The apoptosis array data showed that each drug induced changes in slightly different set of apoptotic proteins: Bardoxolone methyl induced a >2-fold increase in cleaved caspase 3, phosphor-p53 (Ser392) and Bax, suggesting induction of intrinsic apoptotic pathway; Carfilzomib induced changes of >2-fold in HIF-1*α* and p21, suggesting induction of an oxidative stress response;^26, 27, 28^ Digoxin produced a >2-fold increase in Bcl-x, Bcl-2, and p21, also suggesting induction of the instrinsic apoptotic pathway; and Navitoclax at 1 *μ*M induced a >2-fold induction in Bcl-x, Bcl-2, Bax, and p21, and at 2 *μ*M, produced a >2-fold change in cleaved caspase 3. For each combination, the apoptotic related proteins regulated are a combination of those that are regulated by each individual compound, although the fold change for other proteins is also observed: for the combination of Bardoxolone methyl and Carfilzomib, cleaved caspase 3 and HIF-1*α* are upregulated >2-fold, as they did for the individual treatment, but also catalase and Bcl-x are upregulated by >2-fold. For the combination of Navitoclax and Carfilzomib, cleaved caspase 3 and HIF-1α continue to be upregulated by >2-fold, in addition to p21 and catalase. Finally, for the combination of Digoxin and Navitoclax, Bcl-x, Bcl-2, and p21 continue to be upregulated, in addition to cleaved caspase 3, cAIP and p21.

## Discussion

3D spheres are being used to generate *in vitro* models that mimic the cellular heterogeneity and the effects of the microenvironment on tumors, as it is clear that these factors highly regulate tumor growth, metastasis and tumor responses to chemotherapeutic treatment.^2, 29, 30, 31, 32, 33, 34, 35^ The challenge remains how to capture all these complex physiological factors in an *in vitro* model that is reproducible and in a miniaturizable format to be used for HTS of large collections of compounds. We have been developing assays that enable us to compare pharmacological responses with cells growing in standard 2D monolayer mode versus in CSC-enriched cell cultures forming spheres.^13^ Here we report how we have been able to expand this work by developing protocols to generate spheres in wells of a 1536-well microtiter plate, thus enabling large-scale HTS. We have used a library of 1912 chemotherapeutic agents to systematically probe how different growth models of cancer cells respond to the current cancer drugs and those in pre-clinical and clinical development. Our analysis of the compound classes that are most represented in pan-actives and differentially active compounds provide mechanistic insights into common cell death pathways, as well as those cellular pathways that may drive cell proliferation differentially between cells growing as monolayers on plastic, forming spheres or in pre-formed spheres. For example, proteasome inhibitors, BCL2 inhibitors, STAT3 inhibitors, NFkB downregulators, HSP90 inhibitors and PI3K inhibitors appear to induce cell death pathways in all cell growth conditions, and for both cell lines tested. Regulation of the NF*κ*B pathway has been characterized as a key survival pathway in invasive pancreatic CSCs, specifically using the same PANC1 cell line.^36^ The role that STAT3 has in regulating CSCs has been studied in a variety of systems and previous research using prostate CSCs demonstrates that inhibiting STAT3 can decrease the invasive potential of these aggressive cells.^16^

We further tested whether compounds targeting these key cellular pathways would synergize with each other in their cytotoxic effect in spheres, the most resistant cell growth mode, and whether these synergistic effects would reproduce in the different growth modes and cancer cell types. Indeed, when selected compounds representing each enriched target class were tested in pairwise dose-response matrices, significant synergies were detected between them. Interestingly, some of the synergistic effects were stronger in sphere cultures, which illustrate the concept that although single-agent responses may be similar in different growth modes, the synergistic effects may be different in magnitude between in each growth mode. We further confirmed the apoptosis enhancing effects of the combinations of Carfilzomib and Bardoxolone, Navitoclax and Carfilzomib, and Navitoclax and Digoxin in larger sphere cultures of the PANC1 cells. Apoptosis protein arrays showed that each compound induces apoptosis by regulating different proteins, and that the enhancement effects in cell death seen for the combinations are most likely due to additive effects by regulation of these different mechanisms rather than potentiation of the same mechanisms of action by the combination of two compounds. Using publicaly available pharmacokinetic data,^37, 38, 39^ we determined that the combination between Navitoclax and Carfilzomib occurs at doses of each compound that are achievable *in vivo*, and therefore this combination is of high interest to evaluate in animal models of pancreatic cancer.

## Materials and methods

### Cell lines and reagents

PANC1 human pancreatic cancer and SN12C human kidney cell lines were obtained from ATCC (Manassas, VA, USA) and cultured according to the ATCC's instructions (Cell Line Verification Test Recommendations, ATCC Technical Bulletin No. 8 (2008)). SCM was prepared as previously described.^14^ The positive control compound bortezomib was purchased from Selleck (Riverside, CA, USA).

### 1536-well cell proliferation assays

Cell proliferation assays were conducted in sterile, tissue culture treated 1536-well white solid bottom tissue plates (catalog number 789173-F, Greiner Bio-One, Monroe, NC, USA). The 2D monolayer cell proliferation and cells forming spheres protocols were as described previously.^13^ Briefly, for 2D monolayer cell growth assay, 500 cells per well were seeded in 5 *μ*l DMEM (PANC1) or RPMI (SN12C) containing glutamine supplemented with 10% FBS and 1X penicillin/streptomycin, using a Multidrop Combi Reagent dispenser and a small pin cassette (Thermo Scientific, Fisher Scientific, Fair Lawn, NJ, USA). After overnight incubation, 23 nl of compound solution in DMSO was transferred using a Kalypsys pintool. The plates were then covered with stainless steel Kalypsys lids and placed into an incubator at 37°C, with 5% CO_2_ and 95% relative humidity. The plates were incubated for 48 h and then 3 *μ*l of CellTiter-Glo reagent from Promega (Madison, WI, USA) was added using a BioRAPTR (Beckton Coulter, Brea, CA, USA). Plates were incubated for 30 min at room temperature, spun at 1000 r.p.m. and relative luciferase units (RLUs) were quantified using a ViewLux (PerkinElmer, Waltham, MA, US). For cells forming spheres, spheres were first grown in a T75 ULA flask (Corning, New York, NY, USA; catalog number 3814) in 50 ml of SCM media with 5000 cells per ml for 7–14 days. Spheres were then dispersed with Trypsin and spun, and the cell pellet re-suspended in the required amount of SCM media to seed 500 cells in 5 *μ*l per well of a 1536-well white solid bottom tissue plates (see above) using a Multidrop Combi Reagent dispenser and a small pin cassette (Thermo Scientific, Fisher Scientific). SCM contained 10 ng/ml human bFGF (Sigma F0291, St. Louis, MO, USA), 20 ng/ml human EGF (Sigma E9644) and 0.4% BSA (Sigma A9418) supplemented with 1X insulin transferrin selenium (Gibco 51300-044, Gaithersburg, MD, USA) and 1% KnockOut™ Serum Replacement (Gibco 10828-028). Compound addition and 48-h incubation was done as described above for the 2D monolayer cell line growth assay. RLUs for each well were normalized to the median RLUs from the DMSO control wells as 100% viability and median RLUs from the no cell control wells as 0% viability.

### 1536-well 3D spheroid assays

3D spheres of PANC1 or SN12C cells were grown in sterile, tissue culture treated 1536-well white solid bottom tissue plates (either catalog number 789173-F, Greiner Bio-One, Monroe, NC, USA or 3836, Corning) as follows: PANC1 or SN12C cells were seeded 500 cells in 8* μ*l of SCM per well using the Multidrop Combi, and the plates then sealed with an breathable adhesive plate seal (Corning, catalog number 6569) and placed in the incubator at 37 °C, with 5% CO_2_ and 95% relative humidity. After 7 days, stainless steel lids from Kalypsys were used to cover the plate to be able to automate the compound addition and detection reagent. For compound addition, 23 nl of compound solution in DMSO was transferred using a 1536 head Kalypsys pintool as described above. The plates were then covered with stainless steel Kalypsys lids and placed into incubator at 37 °C, with 5% CO_2_ and 95% relative humidity for 48 h. In all, 3 *μ*l of CellTiter-Glo® reagent from Promega was added using a BioRAPTR® (Beckton Coulter). Plates were incubated for 30 min at room temperature, spun at 1000 r.p.m. and RLUs were quantified using a ViewLux (PerkinElmer).

### Microscopy of the 3D spheres formed in 1536-well microtiter plates

PANC1 and SNC12 spheres were generated in 1536-well in black, clear bottom microtiter plates (catalog number 3836, Corning) as described above. Once spheres were formed, they were stained with Hoechst dye 33342 (Invitrogen, Carlsbad, CA, USA), at a concentration of 1:1000 and PI (Invitrogen) at a concentration of 1:500 and incubated for 2 h at room temperature. The spheres were then imaged with an IN Cell 2000 (GE Healthcare, Marlborough, MA, USA) using a 10 × 0.45 NA lens. Spheres were imaged with three channels; brightfield, DAPI (350/50 excitation, 455/50 emission), and PI (Texas Red; 579/34 excitation, 624/40 emission) at 50, 100, and 25 ms exposures, respectively. Images were analyzed using GE's IN Cell Developer V1.9.2 analysis software (GE Healthcare). Briefly, the DAPI channel was used to identify two classes of spheroids (large and small) using an intensity segmentation algorithm (objects greater than 255 RFU). Large spheroids were categorized as having an area of 200 *μ*m^2^ or more, whereas small spheroids and individual cells were categorized as having an area of <200 *μ*m^2^. PI objects were also identified with intensity segmentation (>215 RFU) and size criteria (>6 *μ*m^2^). PI-positive objects in within DAPI spheroids were calculated using standard Developer target linking.

### MIPE compound library

The library utilized in these studies is an NCATS internal collection of 1912 small molecules known to modulate oncology targets, pathways and phenotypes, referred to as the MIPE-oncology library (MIPE).^19, 20, 21^ The library includes approved drugs, compounds in clinical development for cancer treatment and compounds in pre-clinical development. In addition, where feasible, the library included several compounds for each target class or cellular mechanism and process. In many cases, a compound may have known polypharmacology, for example, kinase inhibitors, and the intended target for which the compound was developed, was used for target enrichment analysis.

### Quantitative HTS

For the screen, the compounds in the MIPE library were transferred to columns 5–48, and controls were added in columns 1–4 of the 1536-well assay plate. Column 1 contained media only; column 2 contained cells with added DMSO, whereas columns 3 and 4 contained the protease inhibitor bortezomib or the antibiotic salinomycin in DMSO (final concentration 10 *μ*M). Compounds were tested as dose responses starting at a stock concentration of 10 mM (final compound concentration of 46 *μ*M) in DMSO, and diluted threefold, also with DMSO. The library was tested at 11 compound concentrations for qHTS analysis as described previously.^25^ RLU for each well were normalized to the median RLUs from the DMSO control wells as 100% viability, and median RLUs from control wells with media only as 0% viability.

### Hit selection from qHTS

Activity of the compounds from the dose-response qHTS screen was determined based on two parameters: (i) % viability at the maximum concentration of compound tested (MAXR); and (ii) CRC classification from dose-response HTS, in which normalized data are fitted to a four-parameter dose-response curves using a custom grid-based algorithm to generate CRC score for each compound dose response.^23, 25^ CRC values of −1.1, −1.2, −2.1, −2.2 are considered highest quality hits; CRC values of −1.3, −1.4, −2.3, −2.4 and −3 are inconclusive hits; and a CRC value of 4 are inactive compounds. See Supplementary Material for list of MAXR, CRC and log IC_50_ for the compounds screened in all conditions.

### Target enrichment analysis

Given a selection of compounds, we identified the annotated targets for these compounds and computed the enrichment for each target, compared with background, using Fisher's exact test.^40^ For this test, the background was defined as all the targets annotated in the MIPE collection. The *P*-value from the test was adjusted for multiple hypothesis testing using the Benjamini–Hochberg method.^41^

### Target differential analysis

We quantified differential behavior of individual curve fit or HTS parameters (MAXR, IC_50_) between two cell lines (or conditions within a given cell line) in a target-wise manner. For any two cell growth conditions, for each cell line, we collected the parameter of interest for each compound, grouped by target. We only considered those targets for which there were at least three compounds annotated with the target. For the case of the maximum response parameter (MAXR), all compounds tested were considered. For the case of IC_50_, we only considered compounds that exhibited high-quality curve classes (CRC −1.1, −1.2, −2.1 and −2.2). The median values for each parameter were calculated for each target and differences in median value was estimated using the Mann–Whitney test.^42^ The *P*-values from the test were adjusted for multiple hypotheses testing using the Benjamini–Hochberg method.

### Compound combination matrix screening

For combination matrix screen, protocols were as described in references.^19, 20, 21^ Briefly, for the 2D monolayer and cells forming spheres assays, compounds were pre-plated using an acoustic dispenser ATS-100 (EDC Biosystems, Fremont, CA, USA). A total of 5 nl of each compound solubilized in DMSO, as well as DMSO control wells, were dispensed with twofold dilutions to generate a 10x10 dose-response matrix. In all, 5 *μ*l of a cell suspension (500 cells per well) were added directly to the plates immediately after compounds pre-plated. For the pre-formed 3D sphere assay, spheres were pre-formed for 7 days in the 1536-well assay plate (5 *μ*l, 500 cells per well) and compounds were added to the wells with pre-formed spheres using the acoustic dispenser ATS-100, as described above. For the cell proliferation assays, cells were incubated with the compounds for 48 h and 5 *μ*l of CellTiterGlo reagent added, and RLU were measured with a Viewlux after 15-min incubation at room temperature. For the apoptosis assay, spheres were incubated with the compounds for 16 h, and 5 *μ*l of CaspaseGlo reagent added. RLU were measured with a Viewlux after 15min incubation. The initial concentration of each compound was adjusted so that the range included approximately >4-fold and <4-fold the IC_50_. Carfilzomib dilutions started at 500 nM, Bardoxolone methyl at 20 *μ*M, LLL-12 at 50 *μ*M, Navitoclax started at 20 *μ*M and Digoxin at 500 nM. Synergism was scored in a variety of methods but for this work, we chose to use sum of delta bliss negative scores from the Delta Bliss matrix plots to rank possible synergistic pairs.^19, 20, 21, 43^ Briefly, a delta bliss score was computed for each pairwise combination of concentrations, for each pairwise combination matrix of two compounds, and all the negative delta bliss scores were added to obtain the sum negative delta bliss score for each pair of compounds as a measure of synergism. The heat maps and delta bliss maps for all these combination responses in dose-response matrix maps at the following links: PANC1 2D monolayer, https://tripod.nih.gov/matrix-client?assay=2321; PANC1 3D spheres, https://tripod.nih.gov/matrix-client?assay=2322; PANC1 Forming spheres, https://tripod.nih.gov/matrix-client?assay=2323; SN12C 2D monolayer, https://tripod.nih.gov/matrix-client?assay=2324; SN12C 3D spheres, https://tripod.nih.gov/matrix-client?assay=2325; SN12C forming spheres, https://tripod.nih.gov/matrix-client?assay=2326; and PANC1 3D spheres caspaseglo, https://tripod.nih.gov/matrix-client?assay=2901.

### PANC1 spheres in 384-well ULA plate and CaspaseGlo assay

PANC1 cells were seeded in a ultra-low attachment (ULA) 384-well plate (Corning # 3830) with 500 cells/30 *μ*l per well in SCM media, and allowed to grow 5 days to form spheres. Spheres were then treated with either DMSO or single and combination doses of Digoxin, Bardoxolone methyl, Navitoclax and Carfilzomib for 16, 20 and 24 h. In all, 15 *μ*l per well of Caspase-Glo 3/7 reagent (Promega) reagent was added using a BioRAPTR dispenser and the plate was covered in aluminum foil and shaken on a VWR Microplate Shaker for 30 min. Luminescence signal was measured with a ViewLux reader with a 30s exposure time.

### PANC1 spheres in T75 ULA flask and CaspaseGlo assay

PANC1 cells were seeded 5000 cells/ml in SCM media in Ultra-Low attachment T75 flask (Corning # 3814), and allowed to grow 5 days to form spheres. The spheres were treated with compound for 20 h. Spheres were pelleted by centrifugation and cell apoptosis was measured by addition of 100 *μ*l of Caspase-Glo 3/7 reagent (Promega) directly to the pellet, incubation at room temperature for 30 min, and then transfer of 30 *μ*l per well to each of three wells in a 384-well plate, to measure the luminescence signal with a ViewLux reader.

### PANC1 spheres in T75 ULA flask and apoptosis antibody arrays

PANC1 cells were seeded 5000 cells/ml in SCM media in Ultra-Low attachment T75 flasks (Corning # 3814), and allowed to grow 5 days to form spheres. Spheres were treated with DMSO, Bardoxolone methyl 2* μ*M, Carfilzomib 2 *μ*M, Navitoclax 1 *μ*M and 2 *μ*M, Digoxin 1 *μ*M, Bardoxolone methyl 2 *μ*M and Carfilzomib 2 *μ*M combo; Carfilzomib 2 *μ*M and Navitoclax 2 *μ*M combo, Digoxin 1*μ*M and Navitoclax 1 *μ*M combo, for 20 h; then cells harvested by centrifugation, and the pellet washed with cold PBS twice. Cell lysates were prepared using RIPA buffer (Thermo Scientific, Rockford, IL, USA) containing PhosSTOP and cOmplete Protease Inhibitor tablets (Roche #04906845001 and 04693132001, Roche Diagnostics Corp., Indianapolis, IN, USA). Protein quantitation was assessed by the BCA Protein Assay Kit (Thermo Scientific). Apoptosis arrays used were the Human Apoptosis Array kit (R&D Systems ARY009, Minneapolis, MN, USA). In all, 190 *μ*g of protein from each sample were used on all arrays and product protocol was followed. The array membrane was visualized with the chemiluminescence detection system (Thermo Scientific) and Chemidoc MP system was used to quantitate the intensity of each antibody on the array (Bio-Rad, Hercules, CA, USA). Each array contains duplicate dots for each antibody, so the signal was averaged for both dots then normalized to the positive control dots on each array.

## Figures and Tables

**Table 1:** 

*Block ID*	*Compound A*	*Compound B*	*Delta bliss sum negative*					
			*PANC1*			*SN12C*		
			*2D monolayers*	*Forming spheres*	*3D spheres*	*2D monolayers*	*Forming spheres*	*3D spheres*
1	Digoxin	Carfilzomib	−1.1	−1.2	−1.0	−0.1	−0.8	−0.7
2	Carfilzomib	Bardoxolone methyl	−3.7	−1.3	−4.7	−0.9	−0.4	−1.6
3	LLL-12	Carfilzomib	−0.6	−2.9	−2.9	−1.1	−1.3	−1.3
4	Navitoclax	Carfilzomib	−2.9	−4.1	−2.8	−2.6	−5.0	−6.3
5	Digoxin	Bardoxolone methyl	−2.5	−1.0	−3.9	−0.7	−2.1	−0.3
6	LLL-12	Digoxin	−1.8	−2.6	−3.0	−1.4	−1.4	−1.1
7	Navitoclax	Digoxin	−1.2	−4.3	−4.7	−2.0	−7.6	−8.7
8	LLL-12	Bardoxolone methyl	−0.2	−0.6	−1.9	−3.0	−0.9	−1.9
9	Navitoclax	Bardoxolone methyl	−2.5	−2.6	−5.5	−3.5	−2.6	−5.5
10	Navitoclax	LLL-12	−0.1	−5.1	−1.7	−3.3	−4.7	−6.0

## Abbreviations

2D: two-dimensional
3D: three-dimensional
MAXR: % activity at the maximum compound dose tested
CG: cardiac glycoside
CSC: cancer stem cells
CRC: curve response class
HTS: high-throughput screening
MIPE: mechanism interrogation PlatE
PI: propidium iodide
qHTS: quantitative high throughput screening
RLU: relative luciferase unit
SCM: stem cell media
TIC: tumor-initiating cells

## Supplementary Material

Supplementary Figures and Tables

Supplementary Mathews et al. qHTS 3D spheres HTS Data

Supplementary Mathews et al. Target Differential Data
