# Supplementary material for: Large-scale pharmacological profiling of 3D tumor models of cancer cells
Source: Cell Death Dis. 2016 Dec 1;7(12):e2492–. doi: 10.1038/cddis.2016.360 (PMC5261027; doi:10.1038/cddis.2016.360)
Supplement: Supplementary Figures and Tables [file cddis2016360x1.ppt]

## Slide 1
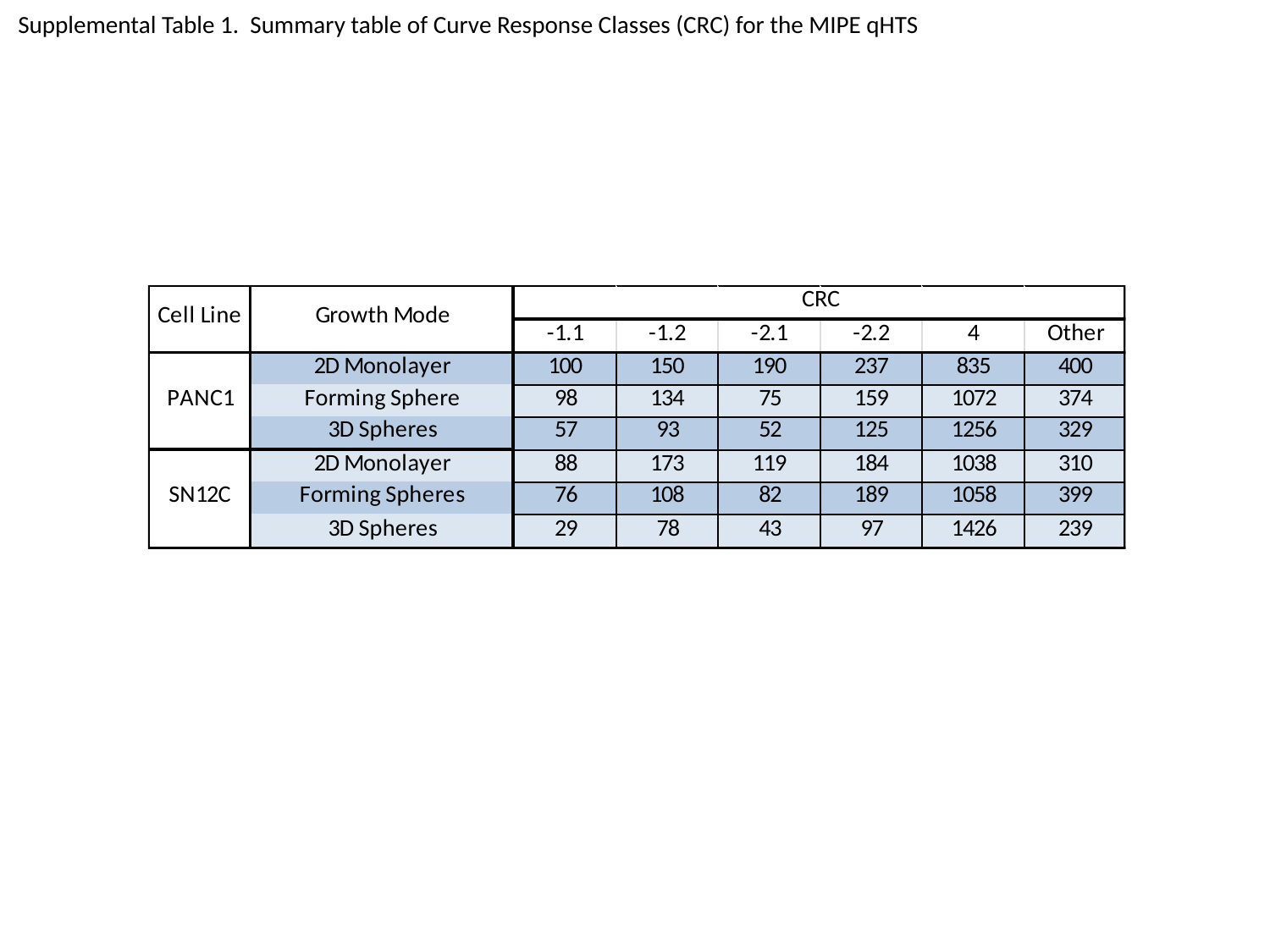

Supplemental Table 1. Summary table of Curve Response Classes (CRC) for the MIPE qHTS

## Slide 2
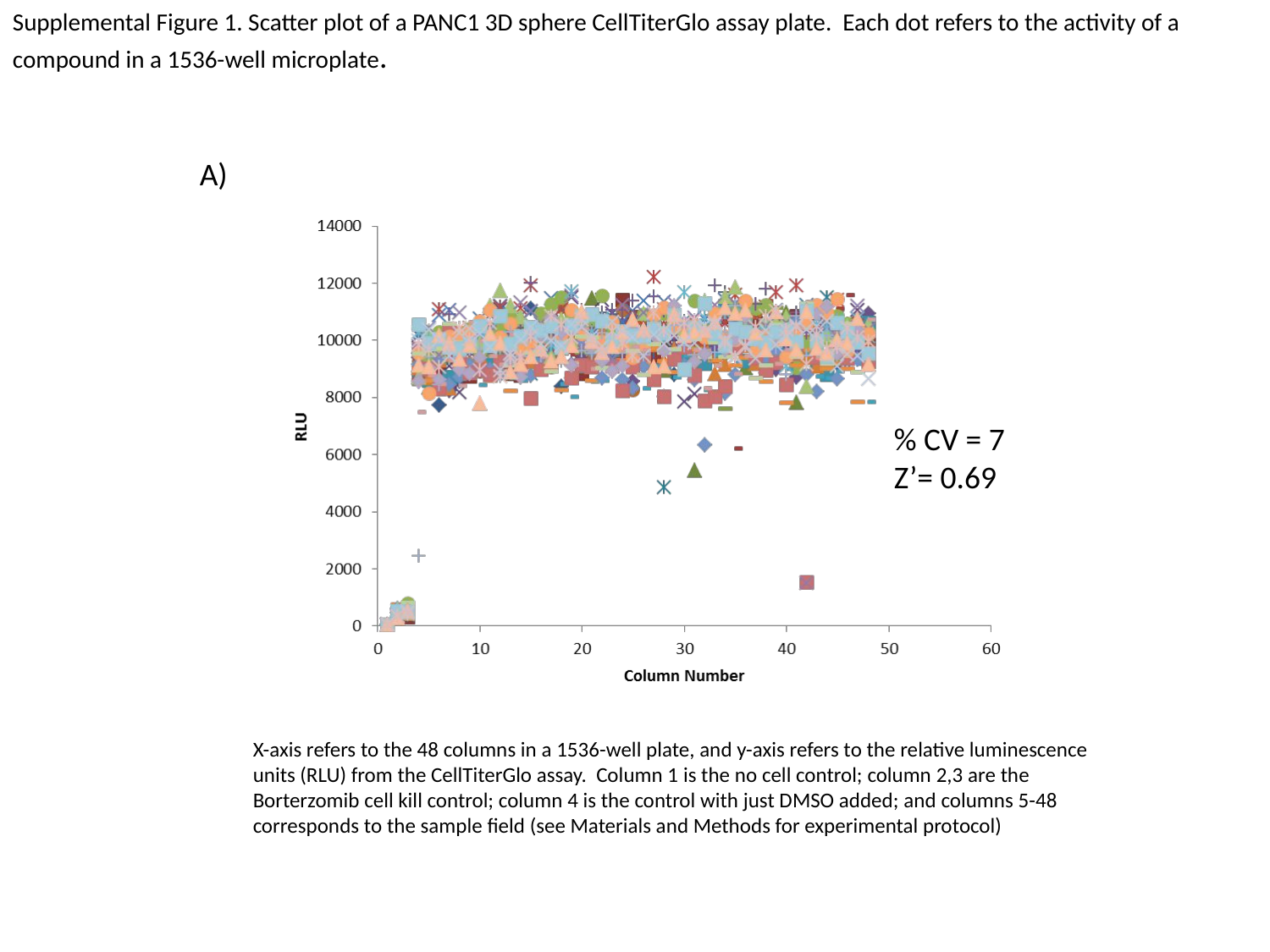

Supplemental Figure 1. Scatter plot of a PANC1 3D sphere CellTiterGlo assay plate. Each dot refers to the activity of a compound in a 1536-well microplate.
A)
% CV = 7
Z’= 0.69
X-axis refers to the 48 columns in a 1536-well plate, and y-axis refers to the relative luminescence units (RLU) from the CellTiterGlo assay. Column 1 is the no cell control; column 2,3 are the Borterzomib cell kill control; column 4 is the control with just DMSO added; and columns 5-48 corresponds to the sample field (see Materials and Methods for experimental protocol)

## Slide 3
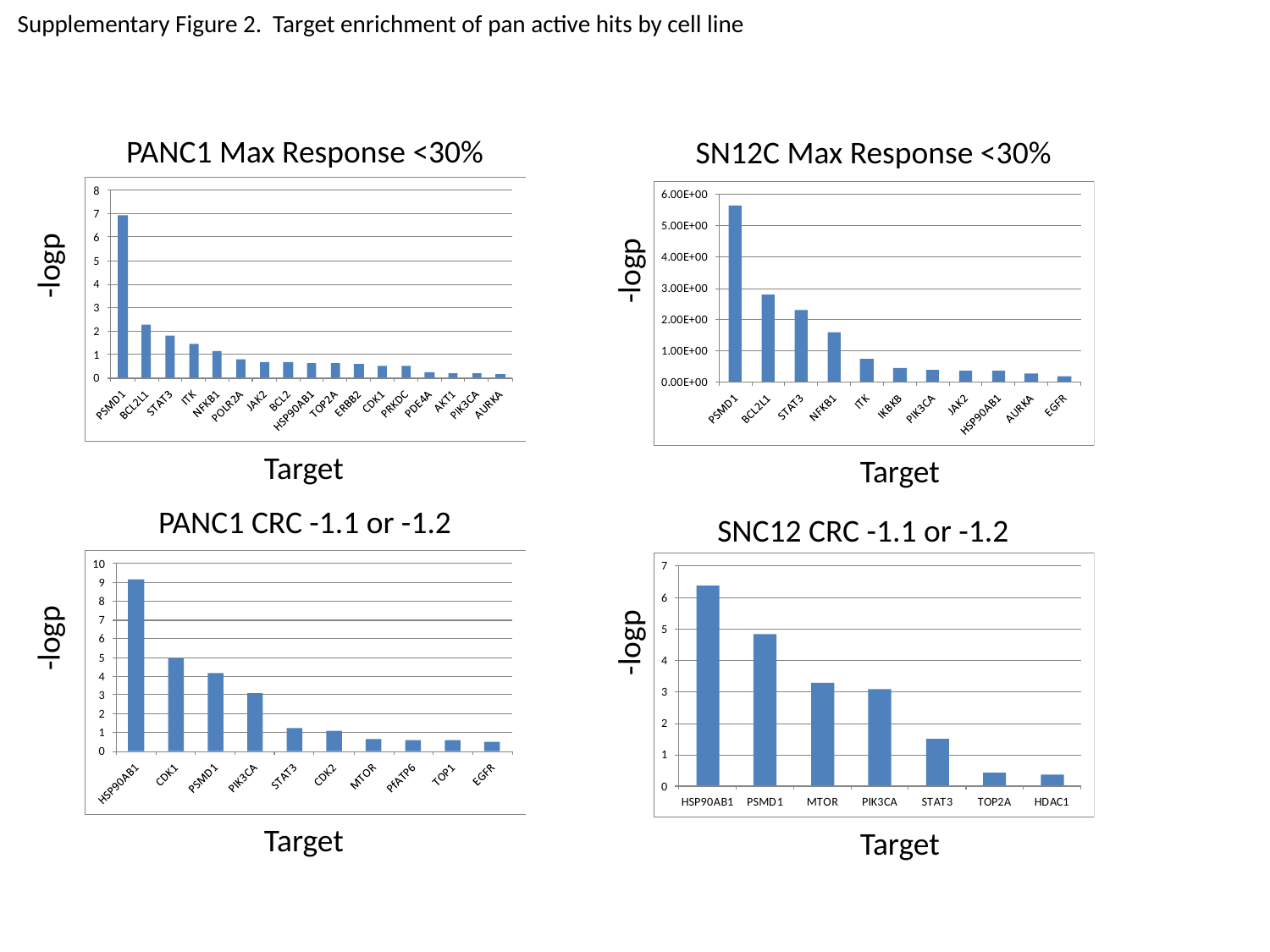

Supplementary Figure 2. Target enrichment of pan active hits by cell line
PANC1 Max Response <30%
SN12C Max Response <30%
-logp
-logp
Target
Target
PANC1 CRC -1.1 or -1.2
SNC12 CRC -1.1 or -1.2
-logp
-logp
Target
Target

## Slide 4
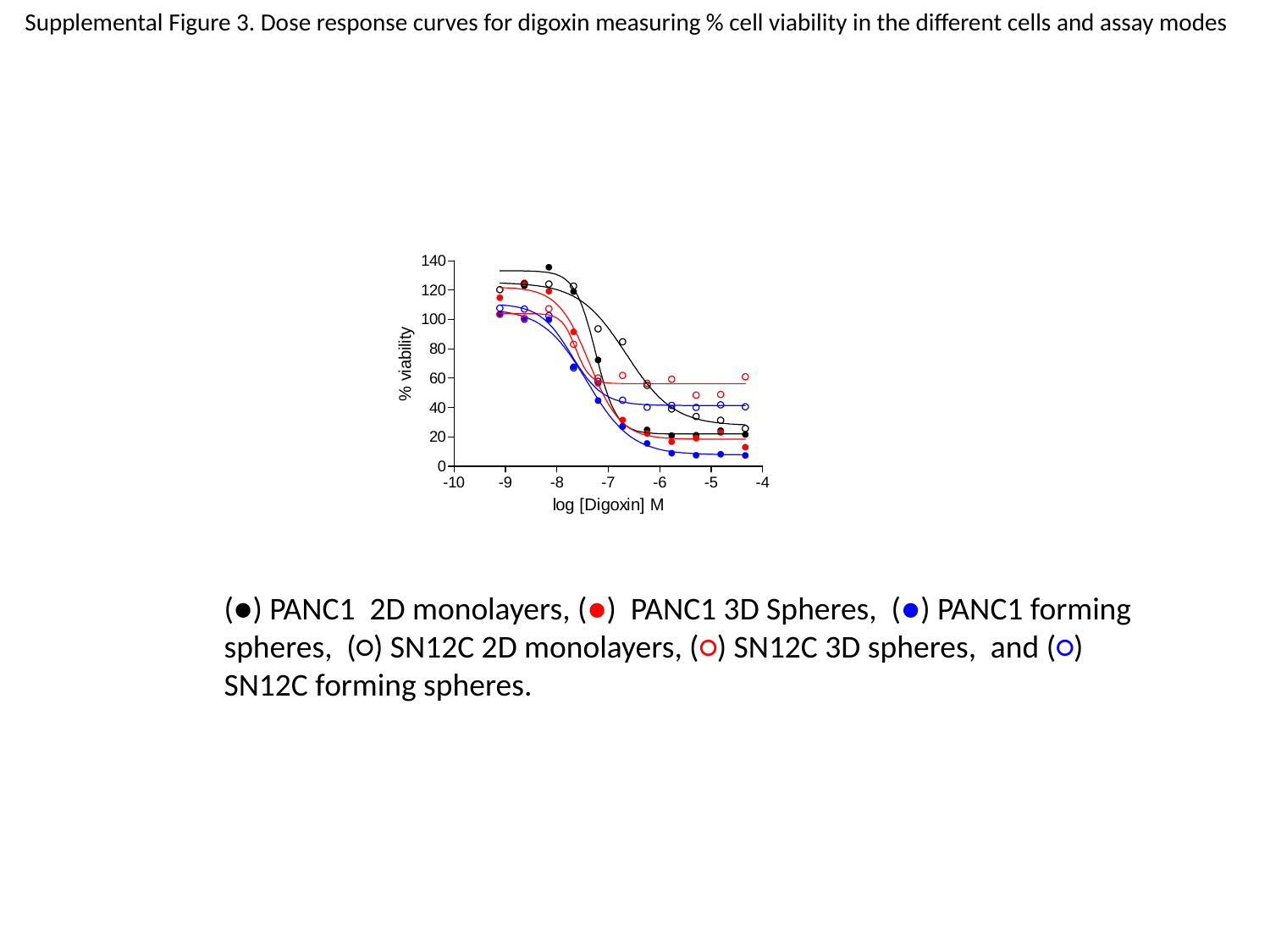

Supplemental Figure 3. Dose response curves for digoxin measuring % cell viability in the different cells and assay modes
(●) PANC1 2D monolayers, (●) PANC1 3D Spheres, (●) PANC1 forming spheres, (○) SN12C 2D monolayers, (○) SN12C 3D spheres, and (○) SN12C forming spheres.

## Slide 5
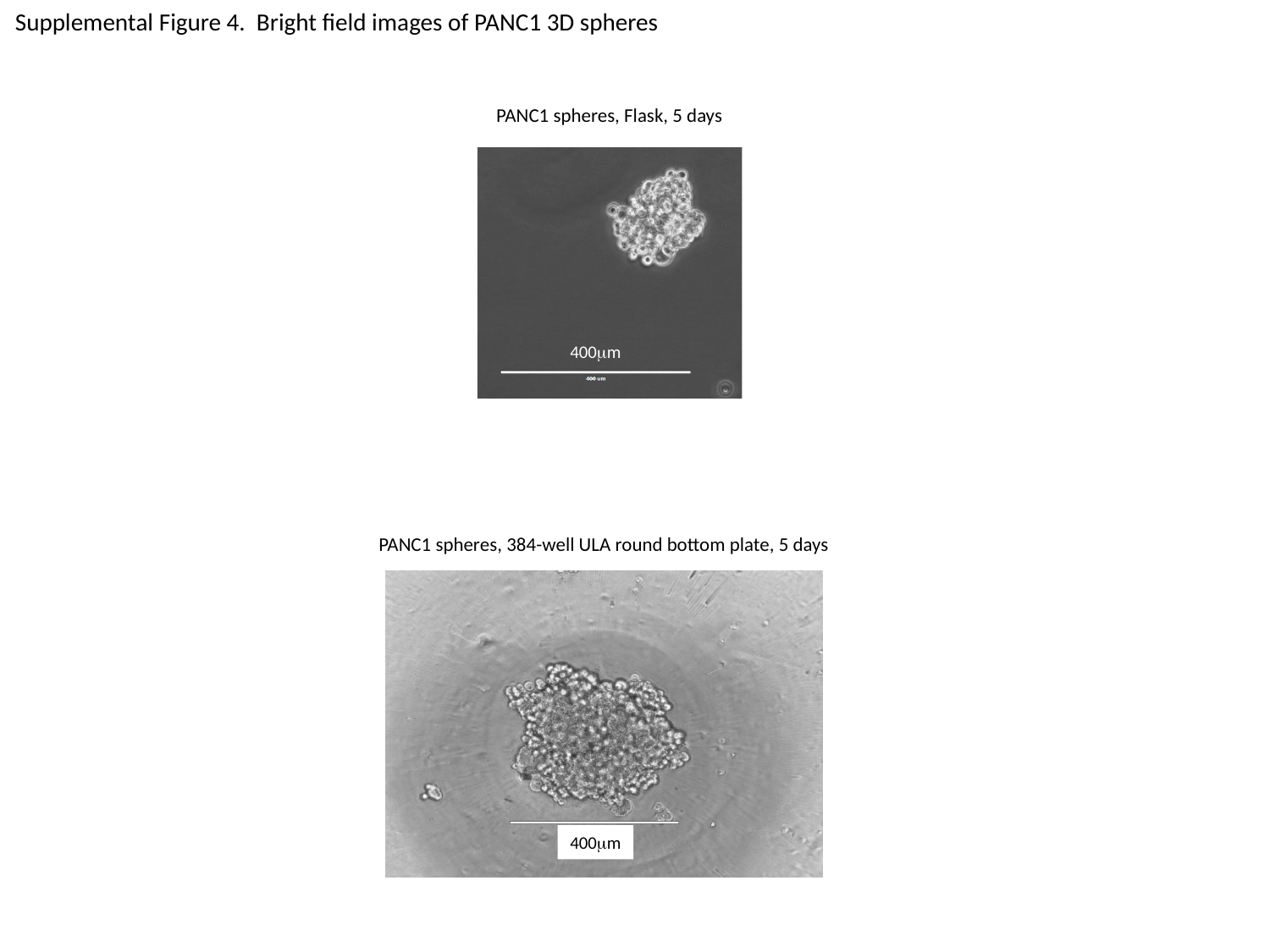

Supplemental Figure 4. Bright field images of PANC1 3D spheres
PANC1 spheres, Flask, 5 days
400μm
1000μm
PANC1 spheres, 384-well ULA round bottom plate, 5 days
400μm

## Slide 6
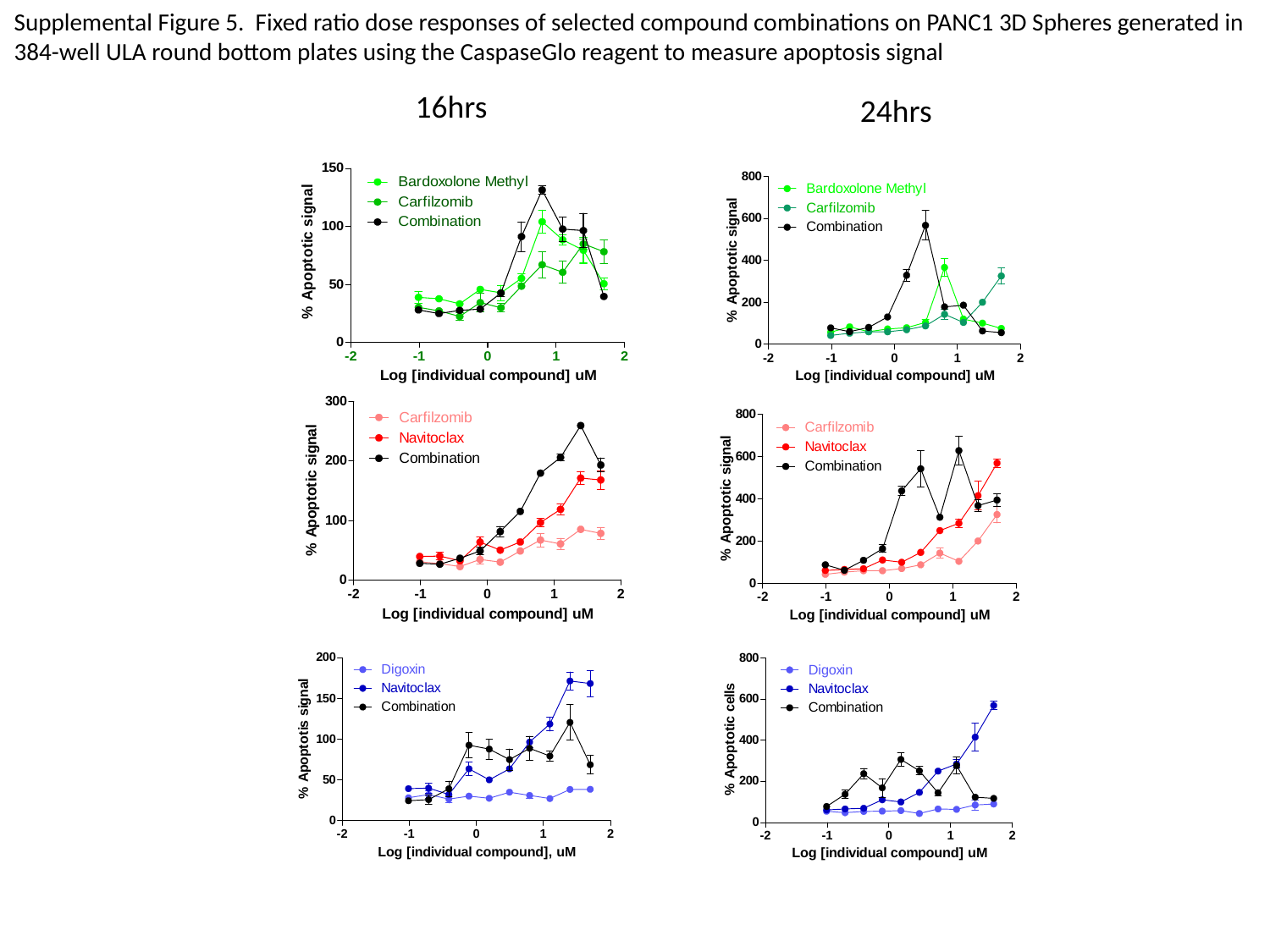

Supplemental Figure 5. Fixed ratio dose responses of selected compound combinations on PANC1 3D Spheres generated in 384-well ULA round bottom plates using the CaspaseGlo reagent to measure apoptosis signal
16hrs
24hrs

## Slide 7
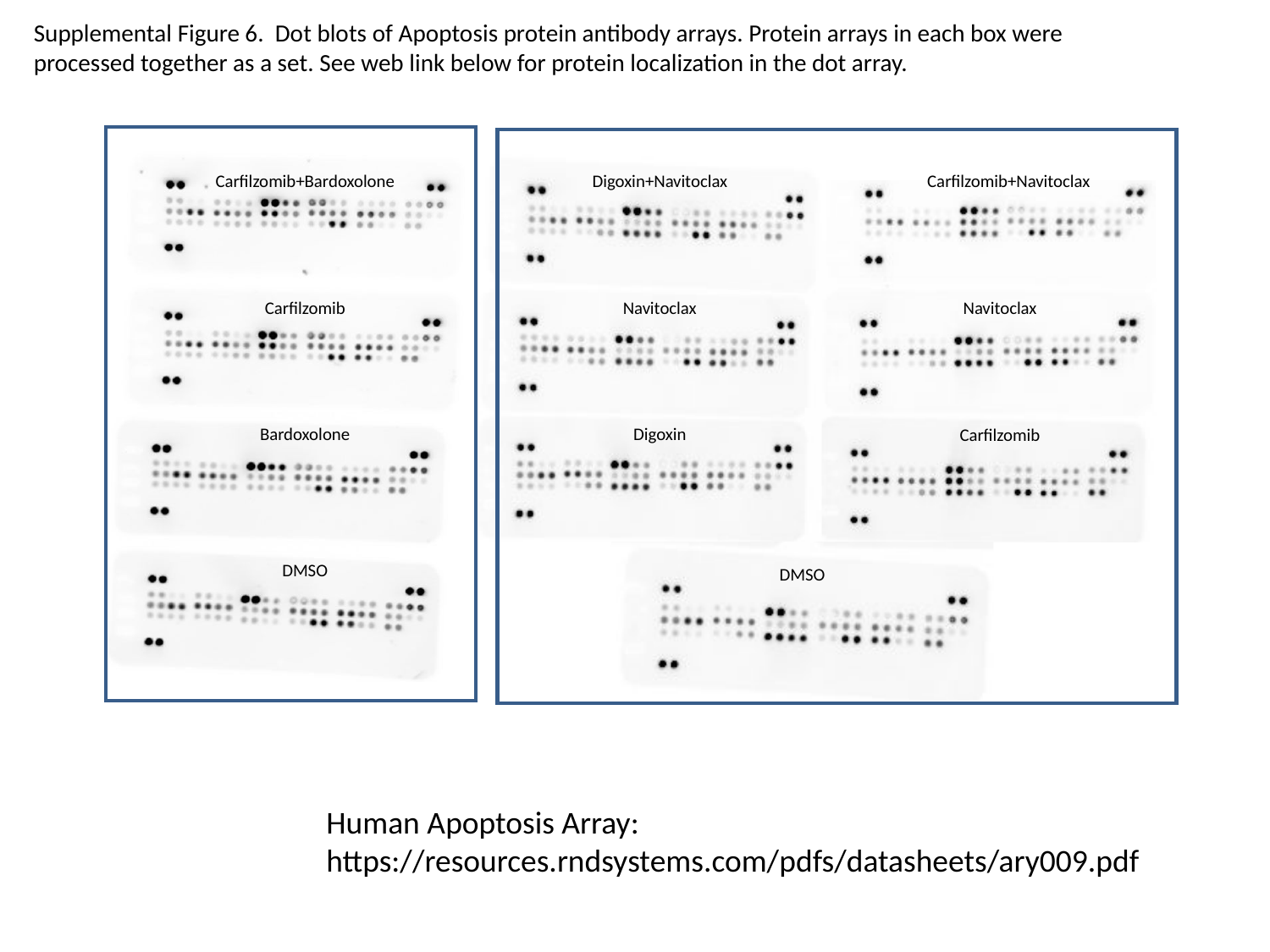

Supplemental Figure 6. Dot blots of Apoptosis protein antibody arrays. Protein arrays in each box were processed together as a set. See web link below for protein localization in the dot array.
Carfilzomib+Bardoxolone
Digoxin+Navitoclax
Carfilzomib+Navitoclax
Carfilzomib
Navitoclax
Navitoclax
Bardoxolone
Digoxin
Carfilzomib
DMSO
DMSO
Human Apoptosis Array:
https://resources.rndsystems.com/pdfs/datasheets/ary009.pdf

## Slide 8
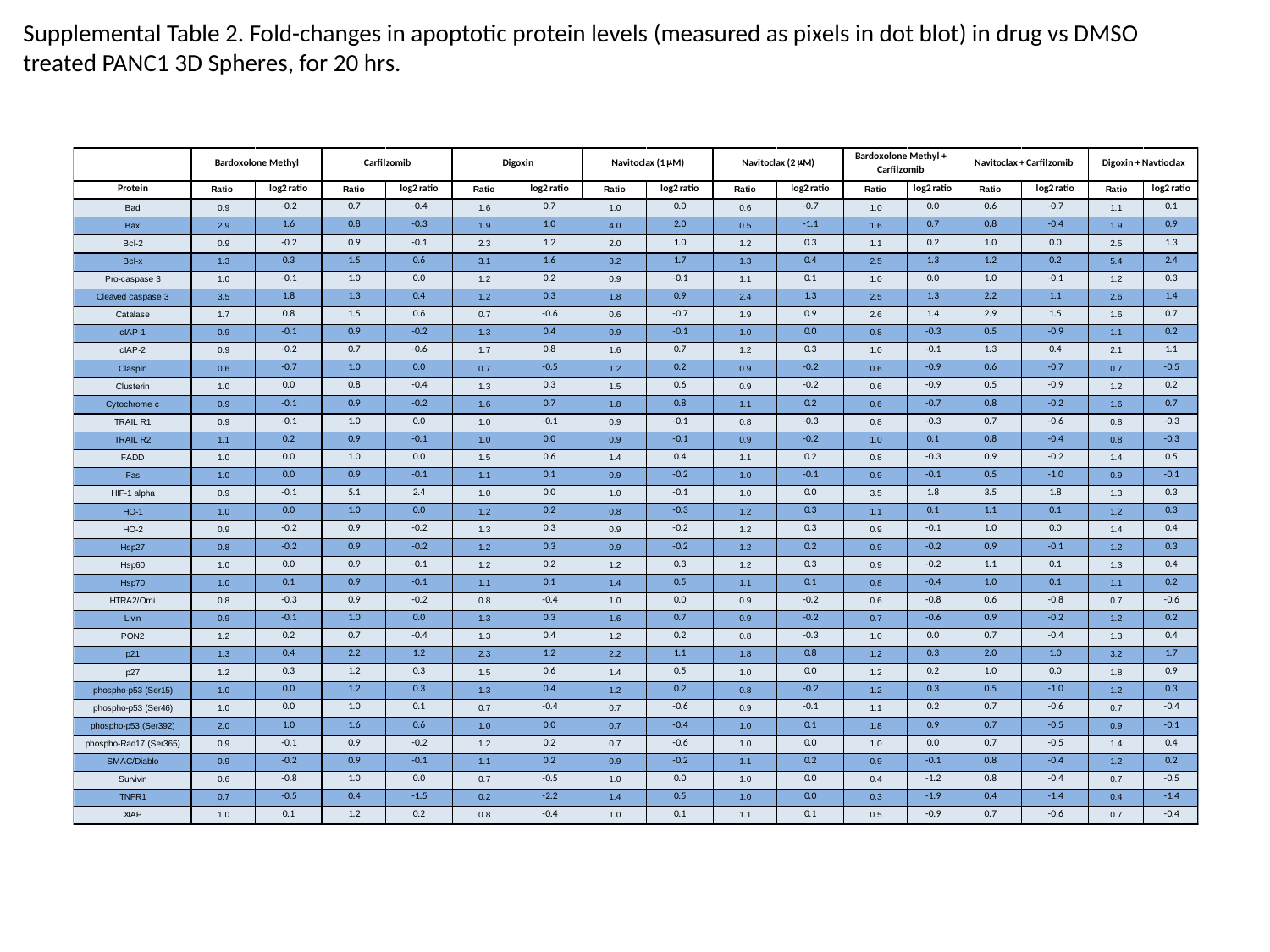

Supplemental Table 2. Fold-changes in apoptotic protein levels (measured as pixels in dot blot) in drug vs DMSO treated PANC1 3D Spheres, for 20 hrs.
